# Operando Visualization of Spatially Heterogeneous Lithium Growth on Ta-Doped LLZO

**DOI:** 10.3390/nano16211414

**Published:** 2026-11-03

**Authors:** Jinchun Guo, Xinwei Zhou

**Affiliations:** School of Optoelectronic Engineering and Instrumentation Science, Dalian University of Technology, Dalian 116024, China; guojinchun@mail.dlut.edu.cn

**Keywords:** solid electrolyte, LLZTO, operando microscopy, lithium deposition

## Abstract

Spatially uneven lithium (Li) growth in garnet solid electrolytes can accompany local mechanical damage. Here, operando scanning electron microscopy was used to follow Li growth on tantalum-doped lithium lanthanum zirconium oxide (LLZTO) under a localized tungsten-probe contact. Independent measurements on fractured and polished surfaces captured both contact-confined growth and visible extension beyond the initial contact region. On a polished surface, remote filamentary features elongated and branched over tens of micrometers. On a fractured surface, Li growth accompanied progressive opening and displacement of neighboring LLZTO regions. Site-specific scanning transmission electron microscopy and energy-dispersive X-ray spectroscopy (EDX) examined a region after extended growth. The maps showed local Ta and Zr signal variations consistent with compositional nonuniformity. Cryogenic three-dimensional EDX revealed similar signal variations in a separate uncycled region. These elemental observations provide local material context for the growth sequences. Overall, the study documents distinct spatial growth modes and their temporal association with mechanical changes. Local composition is identified as a possible contributing factor alongside evolving contact and surface conditions.

## 1. Introduction

Lithium-ion batteries (LIBs) have become a key technology for rechargeable energy storage because of their high energy density and long cycle life [1,2,3]. Growing demand for higher energy density continues to drive improvements in electrode materials and alternative cell designs [4]. Lithium (Li) metal is an attractive anode material because of its high theoretical specific capacity and low electrochemical potential [5,6]. Solid-state batteries (SSBs) offer a promising route to using Li metal anodes while providing opportunities to improve battery safety [7,8]. Their performance depends on fast ion transport through solid electrolytes and reliable contact at solid–solid interfaces [9,10]. Recent assessments emphasize that interfacial stability, reversible Li deposition, and the coupling between electrochemical and mechanical processes remain important challenges [11,12]. Studies of anode-free SSBs further highlight the connections between Li nucleation, growth, stripping, and the chemical and mechanical properties of solid interfaces [13].

Among inorganic solid electrolytes, garnet-type lithium lanthanum zirconium oxide (Li_7_La_3_Zr_2_O_12_, LLZO) has attracted considerable interest because of its fast Li-ion transport [14]. Its conductivity is closely related to crystal structure, Li-site occupancy, and dopant chemistry [15,16]. Tantalum (Ta)-doped LLZO (LLZTO), commonly expressed as Li_7−x_La_3_Zr_2−x_Ta_x_O_12_, provides a means of adjusting the Li content and ionic conductivity through cation substitution. Optimized LLZTO compositions can reach room-temperature ionic conductivities of approximately 10^−3^ S cm^−1^ [17]. In addition to bulk ionic transport, contact between Li metal and the garnet electrolyte strongly affects cell performance. Interfacial modification can substantially reduce the resistance at this solid–solid contact [18]. However, Li penetration through garnet electrolytes can still cause internal short circuits during cycling [19,20]. Understanding where Li growth becomes visible and how it develops relative to the surrounding electrolyte is therefore important for improving the reliability of these materials.

Li intrusion can be influenced by flaws on the solid-electrolyte surface at, or accessible from, the plating-side metal-current-collector/solid-electrolyte contact. Porz et al. identified such flaws as preferred sites for Li intrusion and discussed the stress generated by Li accumulation within them [21]. They proposed that Li accumulation within these flaws generates stresses that drive further crack growth. Synchrotron X-ray tomography has linked pore connectivity in LLZO to its susceptibility to short-circuiting [22]. Operando optical microscopy has revealed several Li penetration morphologies and followed their propagation and reversibility [23]. In situ X-ray imaging of sulfide electrolyte cells has also tracked plating-induced cracks and Li intrusion [24]. Gao et al. used in situ transmission electron microscopy (TEM) to show how mechanically confined Li growth can deform and fracture LLZTO [25]. Combined optical and X-ray microscopy has further revealed local strain and dislocations associated with dendrite branching in single-crystalline LLZTO [26]. These studies establish a close connection between localized Li growth and the mechanical evolution of the surrounding electrolyte.

Microprobe-based scanning electron microscopy (SEM) has extended these studies to local electrode contacts. Zhao et al. constructed a cell inside a focused ion beam (FIB)-SEM system and observed Li filling, movement, and extrusion along cracks in LLZTO [27]. McConohy et al. statistically examined Li intrusion using operando microprobe SEM and found that increasing the probe contact force reduced the characteristic Li-deposit diameter at failure [28]. These studies show that contact mechanics, deposit geometry, and the response of local defects are important parts of microprobe measurements.

Local electronic transport provides another possible contribution to Li deposition within solid electrolytes. Using operando neutron depth profiling, Han et al. observed Li accumulation inside LLZO during plating and associated this behavior with electronic conduction through the electrolyte [29]. Liu et al. found substantially reduced bandgaps at selected grain boundaries in aluminum-doped LLZO. Their in situ TEM observations and electron energy-loss spectroscopy showed Li formation along grain boundaries during biasing [30]. Zhu et al. combined operando Kelvin probe force microscopy with electron-injection experiments to examine electron accumulation and Li deposition at LLZO grain boundaries [31]. These findings identify local electronic properties as another factor in the spatial distribution of Li formation.

Recent studies have further examined the location and sequence of Li deposition. Siniscalchi et al. combined plasma-FIB serial sectioning with magnesium tracing to investigate subsurface Li deposits in LLZTO. They linked isolated subsurface deposition with the formation of surface spallation cracks [32]. Liu et al. used isotope tracer-exchange nuclear magnetic resonance (NMR) and magnetic resonance imaging (MRI) to study dendrite formation in LLZO cells [33]. The NMR results distinguished nonuniform interfacial plating from local Li^+^ reduction at grain boundaries. In situ MRI showed that these processes contributed at different stages of cycling, with early growth near the electrodes followed by increased Li formation in the electrolyte interior. These measurements highlight the importance of combining spatial information with the time evolution of Li growth.

The mechanical environment and local chemistry also affect how Li deposits develop. Cui et al. applied in-plane biaxial compression to LLZTO to deflect dendrite propagation and delay short-circuiting [34]. This allowed internal initiation to be examined during prolonged cycling. Under extreme cycling conditions, localized deposits at grain-boundary junctions and pores or inclusions were identified as Li initiation sites. Xu et al. introduced silver ions into the surface of a Ta-doped garnet electrolyte by annealing an ultrathin silver coating [35]. Operando microprobe SEM and nanoindentation linked improved resistance to Li intrusion to surface toughening and increased tolerance of mechanical defects. The authors found little change in surface electronic conductivity or Li wettability, illustrating a chemical–mechanical contribution to the deposition behavior.

A related question is how Li growth extends beyond an initial microscopic electrode contact and how the adjacent electrolyte responds. Fractured and polished surfaces provide different local environments for examining this process. Fracture exposes grain faces, boundaries and steps, whereas polishing produces a more continuous surface with scratches and possible near-surface damage. These differences can change contact geometry, wetting and the sites available for Li accumulation. Contact mechanics and local defects are important in microprobe plating experiments [28,35]. Comparing the two surfaces therefore helps establish how extended growth develops under different surface conditions.

Electron irradiation can also influence Li redistribution in garnet electrolytes [31]. The measurements were therefore conducted using a small aperture and a low measured specimen-current signal. This allows time-resolved observation while limiting irradiation of the electrolyte.

Here, we combine operando SEM with local elemental characterization to examine Li growth on LLZTO under a tungsten (W)-probe contact. Independent measurements on fractured and polished pellets resolve contact-confined growth, remote filament extension and the spreading of an existing deposit. Substrate-referenced image analysis quantifies local boundary displacement and filament extension while tracking visible probe-shaft features. Site-specific scanning transmission electron microscopy (STEM) and energy-dispersive X-ray spectroscopy (EDX) compare regions examined after extended and contact-confined growth. Cryogenic three-dimensional (3D) EDX supplies complementary information from an uncycled region. Together, these measurements connect spatially heterogeneous Li growth with mechanical evolution and local compositional features.

## 2. Materials and Methods

Commercial LLZTO powder (product code: MA-EN-SS-0145) was purchased from Canrd Technology Co., Ltd. (Dongguan, China). with the supplier-specified formula Li_7−x_La_3_Zr_2−x_Ta_x_O_12_ and nominal range x = 0.4–0.7. Bulk Li, La, Zr and Ta contents in the LLZTO powder were measured by inductively coupled plasma optical emission spectroscopy (ICP-OES; Thermo ICP-OES 6300, Thermo Fisher Scientific, Cambridge, UK). Two independent digestions gave a mean Ta/Zr molar ratio of approximately 0.460, corresponding to x ≈ 0.63 when Zr + Ta is normalized to two sites. The analytical results are provided in Tables S1 and S2.

The powder was loaded into a 10 mm diameter die and uniaxially pressed at 350 MPa without any binder using a laboratory hydraulic press (MTI YLJ-24T, MTI Corporation, Richmond, CA, USA). The pellets were then placed in an alumina crucible, buried in sacrificial LLZTO mother powder to suppress lithium volatilization during high-temperature sintering, and covered with a crucible lid. The covered crucible was transferred to a high-temperature box muffle furnace (MTI KSL-1200X, MTI Corporation, Richmond, CA, USA), and the pellets were sintered in air at 1200 °C for 4 h with a heating rate of 5 °C min^−1^. The cooling rate was 4 °C min^−1^. The sintered pellets were manually polished using 400, 800, and 2000 grit sandpapers in an Ar-filled glovebox (SG1200TS, Vigor Tech (Suzhou) Co., Ltd., Suzhou, China). Two types of surfaces were used for operando measurements. Polished pellet surfaces were used directly in some experiments. In other experiments, the pellets were fractured in an Ar-filled glovebox to expose fresh fracture surfaces and individual LLZTO grains. Representative low-magnification SEM images of the polished and fractured surfaces are provided in Figure S1. The polished surface contains abrasion marks, whereas the fractured surface reveals the granular microstructure and local openings. The fractured surface shows a well-sintered granular microstructure with clearly resolved grains. Statistical analysis of 131 grain profiles gives an average apparent equivalent-circle diameter of 7.6 ± 1.8 μm (mean ± SD; Figure S2).

Powder X-ray diffraction (XRD) was performed using a Rigaku SmartLabXE diffractometer (Rigaku Corporation, Akishima, Japan) with Cu Kα radiation (λ = 1.5406 Å) operated at 40 kV and 150 mA. The diffraction patterns were collected over a 2θ range of 10–80° with a step size of 0.01° and a scan rate of 8° min^−1^. The measured XRD pattern of the LLZTO powder is shown in Figure S3 together with the reference pattern of cubic garnet (PDF#80-0457). The major diffraction peaks are in good agreement with the reference pattern and can be indexed to the characteristic reflections of the cubic garnet structure, confirming that the LLZTO powder possesses the expected cubic garnet phase.

The stage/Li/LLZTO assembly was completed in the Ar-filled glovebox. Air exposure during transfer to the SEM was less than 20 s, with the laboratory relative humidity below 20%. These short, low-humidity transfer conditions were used to limit moisture-assisted surface reactions [36].

**Figure 1:**
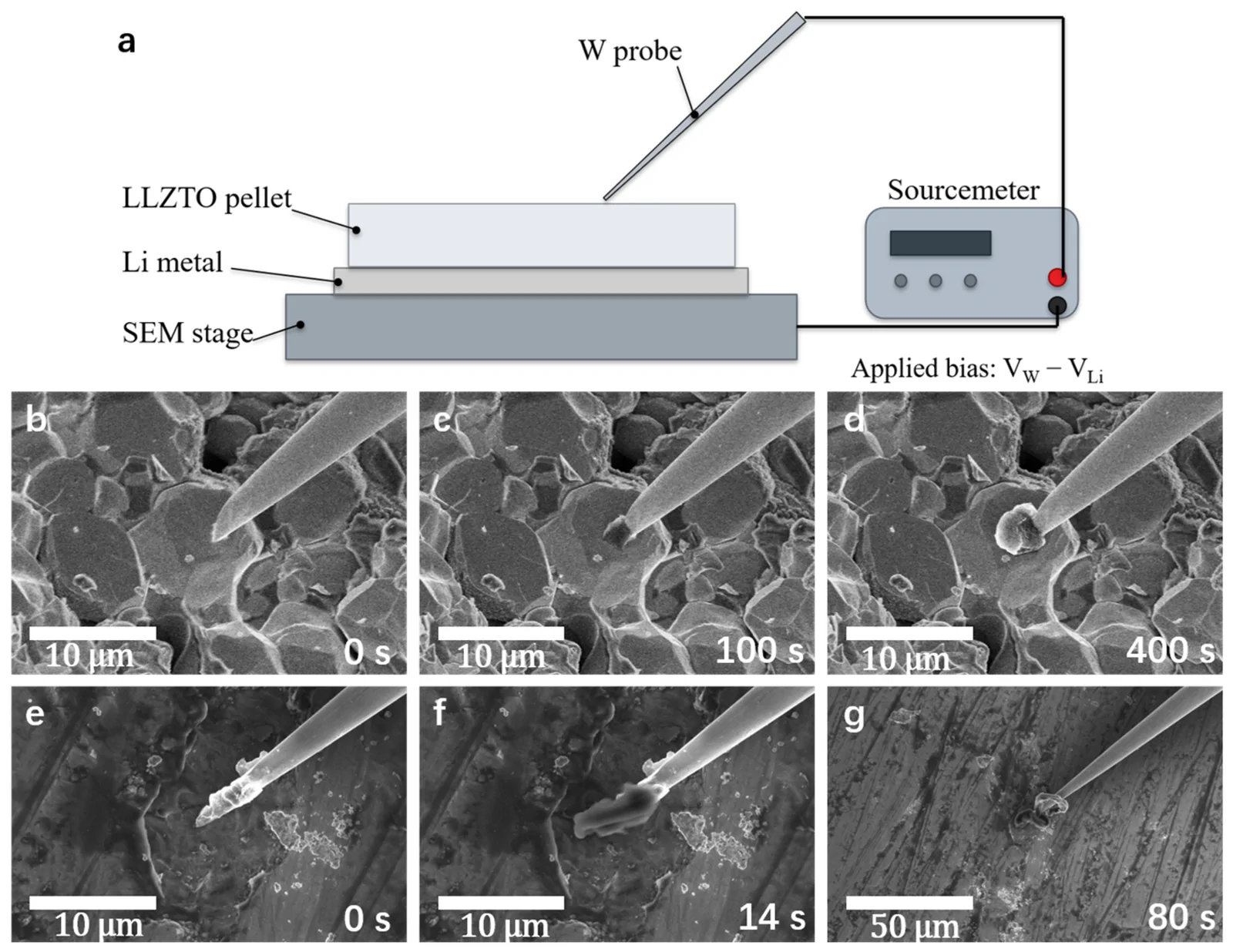
Operando SEM configuration and representative Li deposition on LLZTO. (**a**) Measurement setup. (**b**–**d**) Fractured LLZTO surface at −0.5 V. (**e**–**g**) Polished LLZTO pellet at −1.0 V. The scale bars are 10 μm in panels (**b**–**f**) and 50 μm in panel g.

Operando Li deposition was observed using a ZEISS Nvision 40 FIB-SEM (Carl Zeiss NTS GmbH, Oberkochen, Germany). The experimental configuration is shown in Figure 1a. Li metal was placed on the SEM stage and served as one electrode. The LLZTO pellet was placed in direct contact with the Li metal. A tungsten probe was brought into contact with the exposed LLZTO surface and served as the other electrode. A Keithley 6430 sub-femtoamp remote sourcemeter (Keithley Instruments, Inc., Cleveland, OH, USA) was used to apply a constant voltage. The applied bias is defined throughout this work as

*V*_applied_ = *V*_W_ − *V*_Li_, (1)

where *V*_W_ and *V*_Li_ are the potentials of the W probe and Li metal, respectively. The local W-probe/LLZTO contact condition varied between individual measurements. The applied bias was therefore adjusted empirically to obtain a Li deposition rate suitable for operando imaging.

All operando SEM observations were performed at an accelerating voltage of 5 kV. A 10 µm objective aperture was used to limit electron irradiation. The specimen-current signal measured separately with the stage-current monitor was approximately 10 pA. The same low-current imaging approach was used in our earlier in situ Ge-lithiation study [37]. Our results showed these imaging conditions could limit beam-induced Li growth. The chamber pressure during imaging was 7.01 × 10^−7^ mbar.

The growth videos were analysed using their recorded frame sequence and the calibration of one frame per second of experimental time. Frames at unchanged magnification were registered by normalized template matching of four substrate patches outside the evolving region. The median patch displacement was removed, and the remaining patch scatter was retained as a registration diagnostic.

Local LLZTO boundary motion was measured from grayscale profiles across a fixed 25-row band. The descending intensity gradient after Gaussian smoothing located the outer edge, and its change from the initial image gave the projected displacement. The image calibration was 2 µm per 36 pixels. Uncertainty includes row-to-row variation, image localization, registration scatter and scale reading. The reported measure is the displacement of the selected outer boundary, distinct from the width of the partially occupied central opening.

The projected span of the principal remote filament was measured as the separation between its selected visible extremities at four times in a fixed-magnification interval. Two persistent W-shaft features were tracked relative to the registered substrate. The calibration was 10 µm per 90 pixels. Endpoint uncertainty was estimated from three pixels per endpoint and one pixel in scale-bar length. Distances are image-plane projections. The source frames, annotation coordinates and numerical results are supplied with the supplementary analysis files.

STEM and EDX were performed using an Talos F200X microscope equipped with a Super-X silicon-drift detector system (FEI Company, Hillsboro, OR, USA) at 200 kV. Specimens from the extended-growth and contact-confined-growth regions were prepared by FIB lift-out. Surface Li at the extended-growth location was first removed mechanically with the W probe. STEM–EDX interpretation is limited to La, Zr and Ta. These are framework cations with much lower mobility than Li, so long-range redistribution of these heavier cations is not expected during preparation [38].

EDX mapping was acquired by repeated scanning with a 1 µs pixel-time setting, 20-fold pixel averaging and a line-average value of 1 (nominally 20 µs per pixel per scan visit), with the mapped area adjusted to the selected region. La Lα, Zr Lβ and Ta Lα intensity maps were exported using ESPRIT software (version 1.9; Bruker Nano GmbH, Berlin, Germany). The displayed signals represent characteristic X-ray intensity.

Three-dimensional EDX characterization was performed on an uncycled LLZTO pellet using a Helios 5 UX FIB system equipped with a CryoMAT system (Thermo Fisher Scientific, Hillsboro, OR, USA). The sample was maintained at approximately −140 °C during data acquisition to reduce cumulative heating and Li-related changes in the matrix during repeated milling and EDX acquisition. Serial FIB milling was performed with a nominal step size of 50 nm. EDX maps were collected after each milling step at an accelerating voltage of 5 kV. A total of 73 serial datasets were acquired and used to reconstruct the three-dimensional distributions of La, Ta, and Zr. The same channel thresholds and normalization procedure were applied to the La, Ta and Zr reconstructions.

## 3. Results

Figure 1b–d and Video S1 show Li deposition on a fractured LLZTO surface at −0.5 V. At 0 s, no obvious deposit was visible at the W-probe/LLZTO contact. Li became visible around the probe tip by 100 s and formed a larger deposit by 400 s. Within the recorded field of view, growth remained concentrated near the contact, while the surrounding exposed LLZTO showed no comparable visible growth.

Contact-confined growth was also recorded on a polished pellet at −1.0 V (Figure 1e–g and Video S2). The deposit near the probe enlarged between the initial frame and 14 s. The magnification was subsequently reduced, giving a wider view at 80 s. Li remained concentrated near the probe in this frame, with no comparable growth visible elsewhere in the expanded field. The two sequences provide reference examples of growth confined near the local electronic contact during their respective observation windows.

The assignment of the growing material to metallic Li follows from the electrochemical configuration and its dynamic response. The Li electrode supplies Li^+^ through LLZTO, and cathodic polarization of the W contact supports the reaction Li^+^ + e^−^ → Li. Growth begins near this electronic contact and continues as polarization is maintained. The expanding, folded and filamentary forms are also consistent with Li growth in related garnet microprobe experiments [19,27,28]. These combined observations support identifying the deposits as Li.

The measurements were performed under vacuum without a liquid electrolyte supplying dissolved salts. Together with the short, low-humidity transfer and the continuous response to cathodic polarization, this supports the assignment to Li growth. Thin surface reaction layers may cover deposited Li without changing this interpretation of the growing body.

In this configuration, Li-ion transport through the electrolyte and electron supply through the probe meet near the contact region. The spatial concentration of growth in Figure 1 is consistent with this geometry. Here, contact-confined growth refers to the visible distribution relative to the probe during the recorded sequence. The following experiments show how the visible deposit can instead extend beyond the initial contact region.

**Figure 2:**
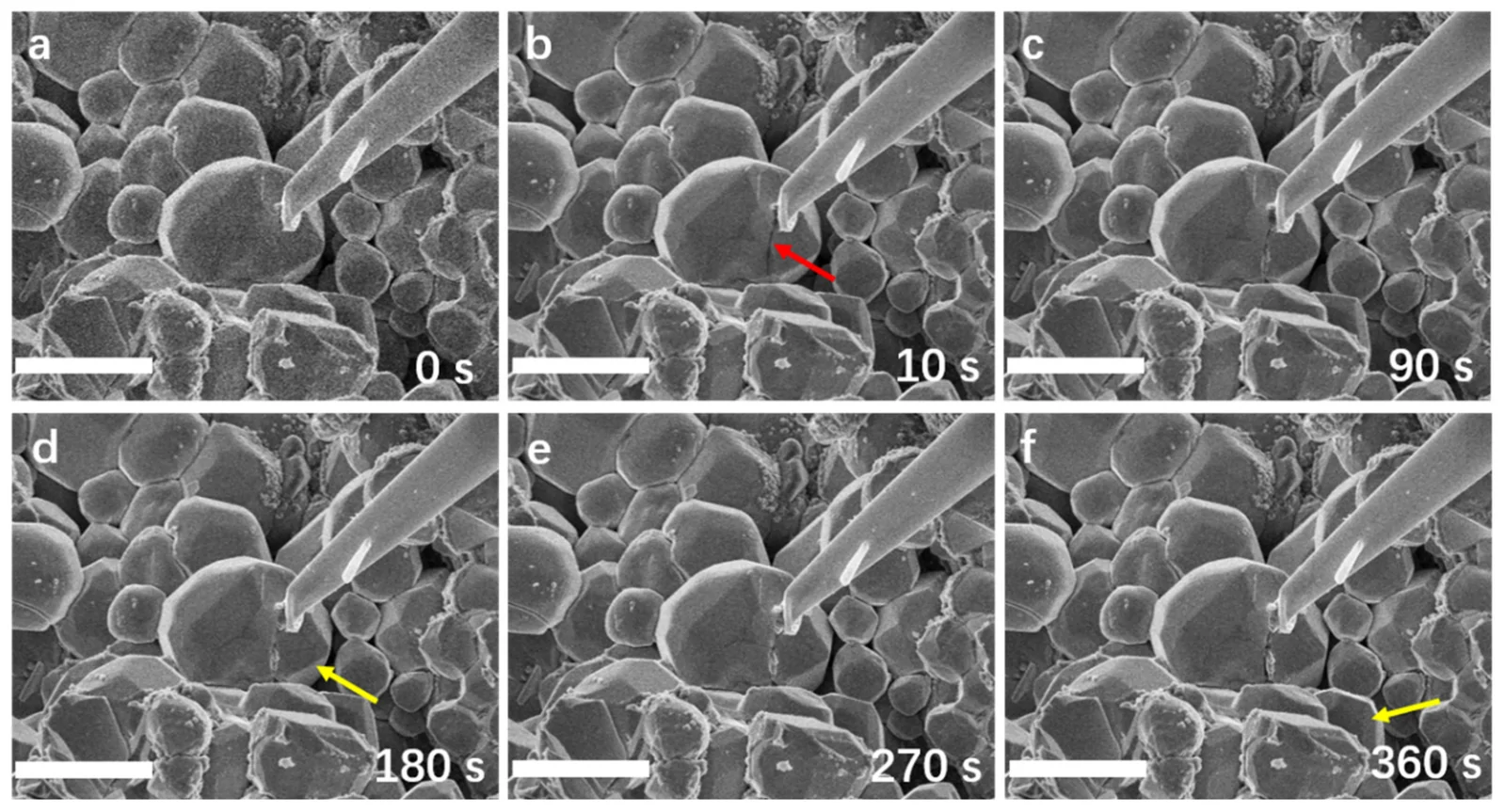
Operando Li growth and local opening on a fractured LLZTO surface. (**a**–**f**) Images at 0, 10, 90, 180, 270, and 360 s under −0.2 V. The red arrow marks a Li feature away from the initial contact; yellow arrows mark opening and displacement. Scale bars: 5 μm.

Figure 2 and Video S3 show growth extending beyond the initial contact on a fractured surface at −0.2 V. By 10 s, a narrow Li feature was visible along the exposed surface below the probe contact, as indicated by the red arrow in Figure 2b. This feature became more pronounced during continued polarization. The sequence records Li growth extending away from the initial contact along a restricted part of the observed surface.

The growth was accompanied by progressive mechanical changes. Between 90 and 180 s, the local opening became wider and a neighboring LLZTO region was displaced. These changes continued at 270 and 360 s. The yellow arrows in Figure 2d,f identify the regions showing opening and displacement. Comparison with the initial frame shows that the local geometry evolved during polarization, rather than simply remaining as an unchanged feature of the fracture surface.

To separate local motion from image drift, the frames were registered against surrounding substrate features (Figure S4). The selected outer LLZTO boundary shifted by approximately 0.37 µm at 90 s, 0.57 µm at 180 s and 0.98 µm at 360 s relative to the initial frame. The final displacement is approximately 1.0 ± 0.2 µm using a conservative image-measurement uncertainty. This directly quantifies local projected motion during Li growth.

The simultaneous growth and displacement are consistent with Li accumulation exerting a mechanical load on neighboring LLZTO. As the local geometry changes, it creates space for further Li accumulation and changes nearby contact conditions. Related microcell experiments have directly observed Li filling and movement in LLZTO cracks [27]. The present sequence adds a time-resolved measure of the adjacent electrolyte response. The quantitative result concerns the outer boundary; the central opening also contains or is partly obscured by growing Li.

**Figure 3:**
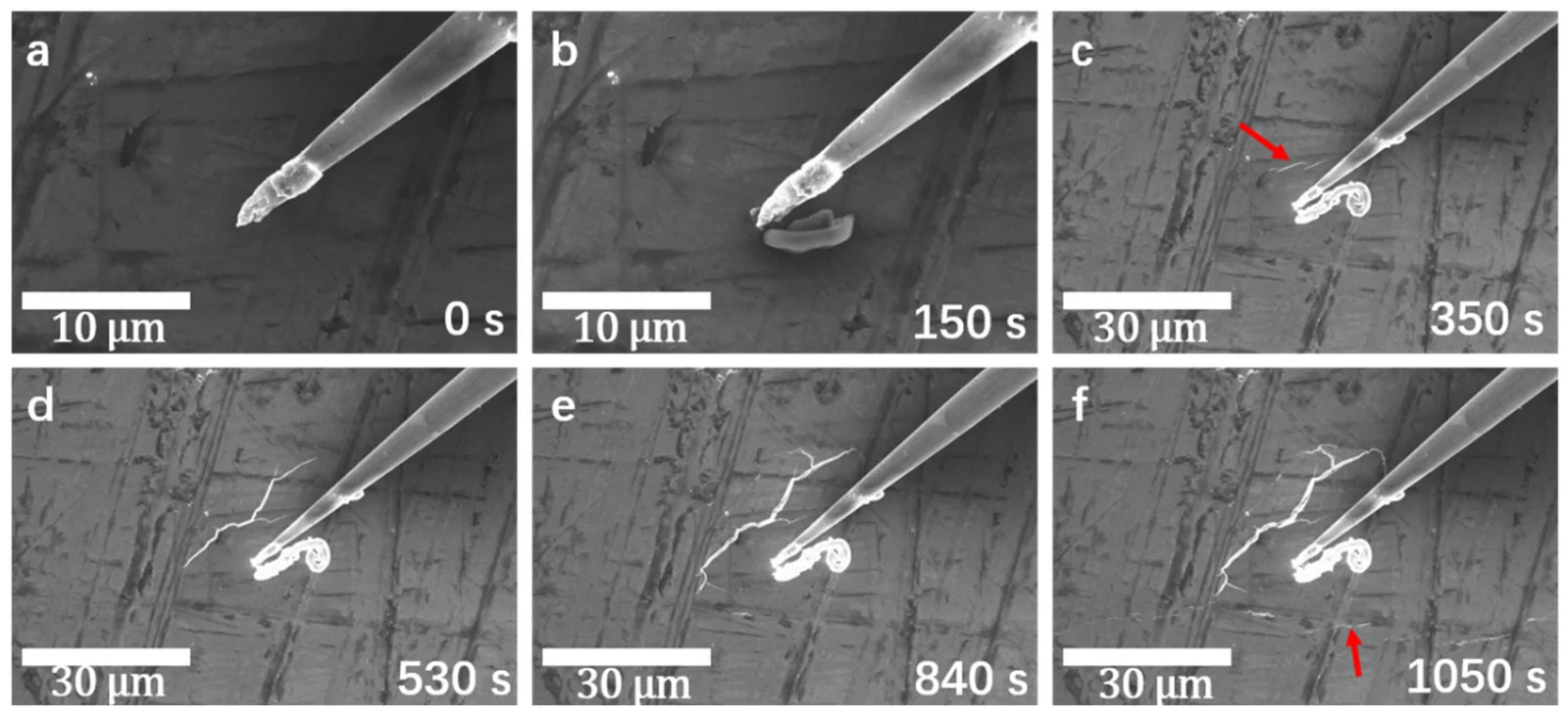
Operando Li growth on a polished LLZTO pellet at −0.5 V. (**a**–**f**) Images at 0, 150, 350, 530, 840, and 1050 s. Red arrows mark visible Li features away from the initial contact.

Extended Li growth was also recorded on a polished LLZTO pellet at −0.5 V (Figure 3 and Video S4). At 150 s, the main deposit remained near the W-probe contact. After the field of view was enlarged, a slender Li feature was visible away from the main deposit at 350 s (Figure 3c, red arrow). The subsequent fixed-magnification frames follow its continued elongation and branching.

The projected endpoint separation of the principal remote filament increased from approximately 16 µm at 350 s to 39 µm at 530 s and 48 µm at 1050 s (Figure S5). Its interval-averaged span increase was approximately 0.046 µm s^−1^. Two persistent features on the visible W shaft moved by approximately 0.3 µm relative to the registered substrate over the same interval. Thus, the tens-of-micrometers extension occurred mainly through growth of the filament relative to the substrate, rather than through a comparable lateral displacement of the probe.

The filament branches and the larger contact deposit followed different visible outlines within the same field. This shows that Li growth can remain spatially concentrated even while its extent increases substantially. The effective electrical contact can also evolve as the deposit enlarges and makes new contact with the electrolyte. Ion supply through the local interface, deposition at newly contacted regions and mechanical redistribution can jointly shape the growth pattern. The reported rate describes the increasing projected span, which is the directly resolved quantity in this sequence.

**Figure 4:**
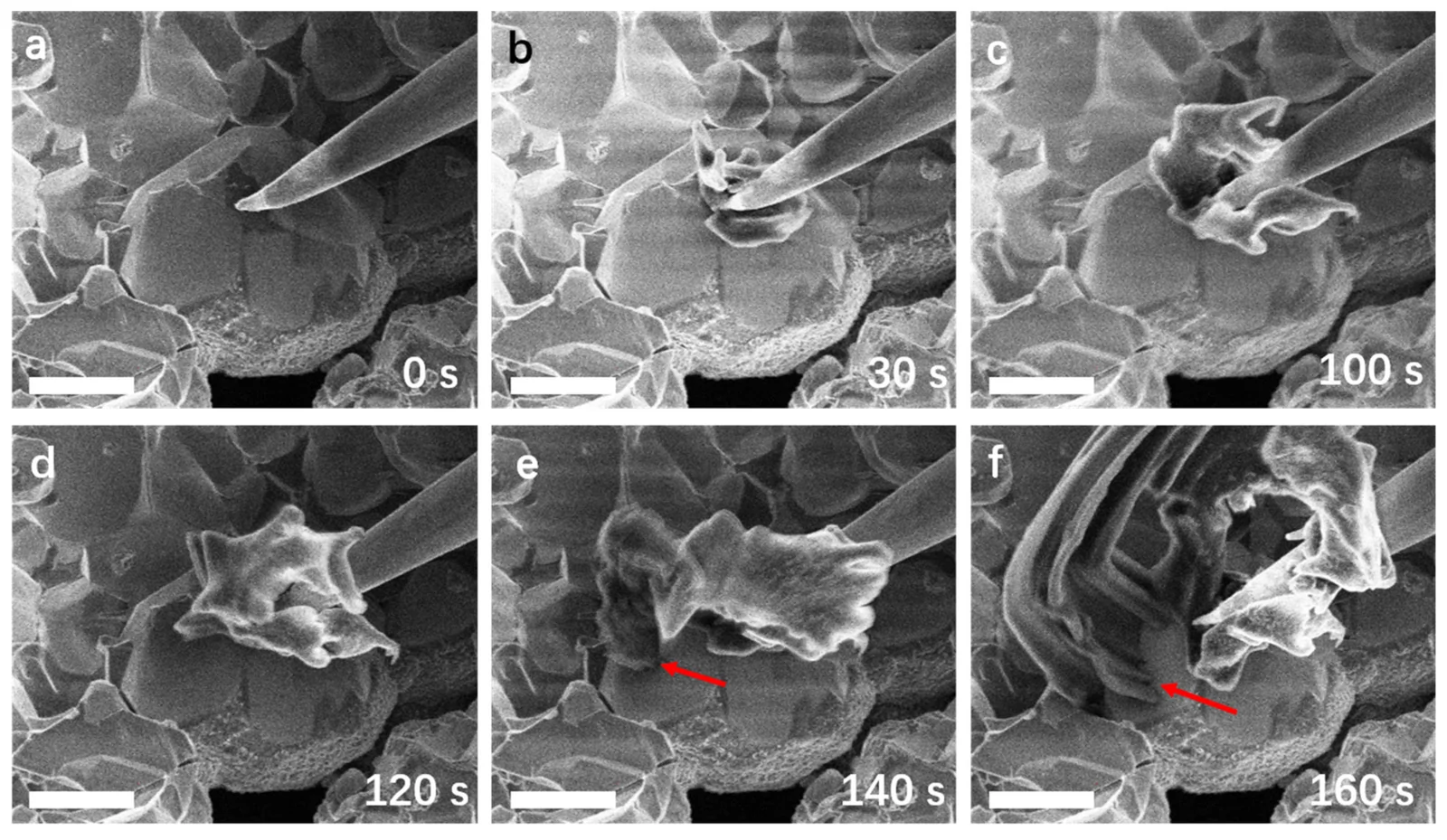
Extension of a Li deposit on a fractured LLZTO surface during a bias step. (**a**–**f**) Images at 0, 30, 100, 120, 140, and 160 s. The bias was −0.5 V for the first 100 s and −2.5 V thereafter. Red arrows mark surface regions reached by the growing deposit. Scale bars: 5 μm.

Figure 4 and Video S5 follow another experiment on a fractured surface. During the first 100 s at −0.5 V, the visible Li deposit developed near the W-probe contact (Figure 4a–c). The bias was then increased to −2.5 V. Rapid enlargement and changes in the deposit shape followed over the next 60 s (Figure 4d–f).

In this sequence, the expanding deposit reached additional parts of the exposed surface. By 140 s, its lower edge extended farther along the LLZTO, and this surface-associated growth became more pronounced by 160 s. The red arrows in Figure 4e,f mark the lower region reached by the deposit. The later images show a large folded structure rather than a uniform coating of the surrounding surface.

This sequence illustrates the extension of an existing Li deposit as its shape and contact with LLZTO evolve. Spreading Li enlarges the metal/electrolyte interface and can establish electronic contact with additional surface locations. Further plating and mechanical redistribution then contribute to the changing outline. The increasingly folded structure shows that growth is not equivalent to the formation of a uniform coating. The LLZTO near the extended deposit was selected for subsequent site-specific microscopy.

**Figure 5:**
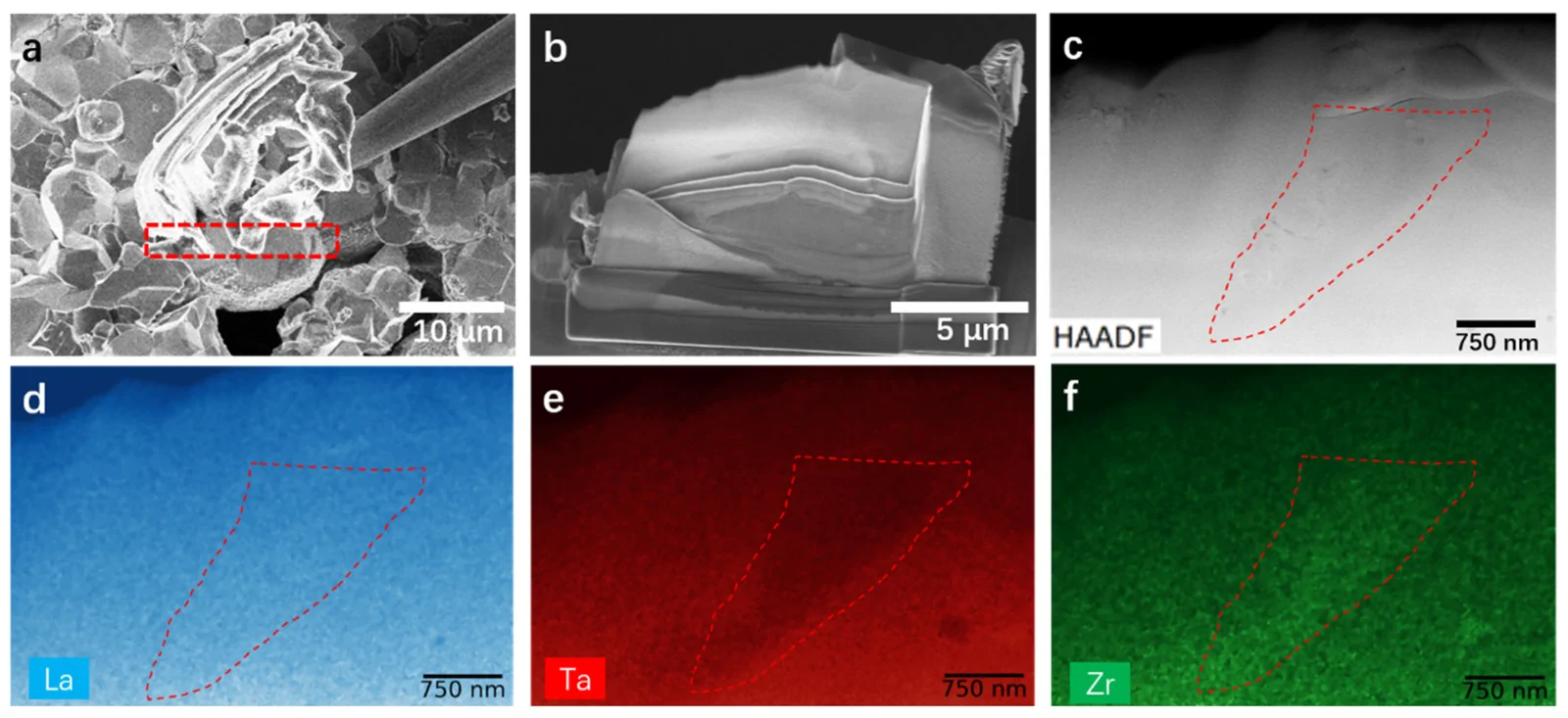
Site-specific STEM-EDX characterization after operando Li growth. (**a**) Location selected for FIB lift-out. (**b**) Prepared TEM specimen. (**c**) HAADF-STEM image. (**d**–**f**) Corresponding La, Ta, and Zr maps. Dashed outlines mark the same selected area in (**c**–**f**).

Figure 5a identifies the selected region after the experiment in Figure 4. Surface Li was mechanically removed using the W probe, and a TEM lamella was prepared from the area marked by the dashed rectangle. Figure 5b shows the FIB-prepared specimen. The high-angle annular dark-field (HAADF)-STEM image in Figure 5c provides the structural reference for the corresponding elemental maps.

Within the outlined area, the La signal varies relatively little, while the Ta signal is weaker and the Zr signal is stronger than in the neighboring material (Figure 5d–f). This contrasting Ta/Zr pattern is consistent with local compositional variation. A separate specimen examined after contact-confined growth provides an additional comparison (Figure S6). Its La, Zr and Ta maps show a more uniform spatial pattern at the displayed scale, without a similarly distinct complementary-contrast region. The comparison concerns the internal spatial pattern of each map, not absolute brightness between separate acquisitions.

The additional specimen provides a useful comparison between two growth regions prepared for STEM in the same way. The distinct Ta/Zr contrast near extended growth, compared with the more uniform elemental distribution after contact-confined growth, supports local cation variation as a material feature worth considering when interpreting the different Li-growth behaviors.

**Figure 6:**
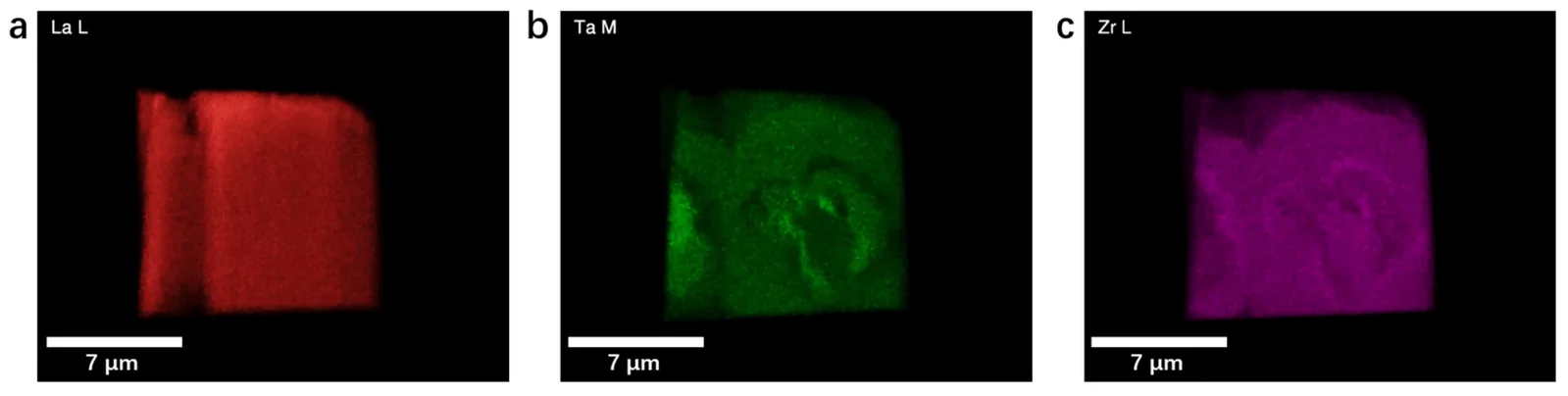
Three-dimensional EDX characterization of an uncycled LLZTO region. Reconstructed distributions of (**a**) La, (**b**) Ta, and (**c**) Zr.

Figure 6 and Video S6 show elemental reconstructions of a separate uncycled LLZTO region. With the same channel thresholds and normalization procedure applied to all channels, local Ta and Zr variations remain visible, while La is comparatively uniform. The comparison concerns the spatial distribution within each channel, not the conversion of different channel brightnesses into elemental concentrations.

The post-test maps, the contact-confined comparison and the uncycled reconstruction provide complementary material information. Ta substitution changes the nominal Li content and can affect ion transport in the garnet framework [17]. Local Ta/Zr variation may therefore contribute to differences in transport or interfacial response. We interpret composition as one contributing factor together with contact geometry and surface condition, rather than as a continuous pathway extending from the probe to every remote feature.

An additional experiment on a polished LLZTO pellet also showed Li growth extending beyond the initial W-probe contact (Figure S7 and Video S7), providing an independent example of the behavior observed in Figure 3. Together with the fractured-surface experiments in Figure 2 and Figure 4, these observations show that spatially extended Li growth can occur on both polished and fractured LLZTO surfaces. The detailed morphology differs between experiments, reflecting differences in local surface topography, contact geometry, and the evolution of the deposit during polarization.

The applied biases also varied among the experiments. Contact-confined growth was observed at −0.5 and −1.0 V in Figure 1, whereas extended growth was observed at −0.2 V in Figure 2 and −0.5 V in Figure 3. In Figure 4, increasing the bias from −0.5 to −2.5 V accelerated the enlargement of an existing deposit. These observations indicate that the magnitude of the applied bias influences the growth rate, but does not by itself determine whether Li remains confined near the probe or extends to more distant locations. Differences in polarization time, initial contact condition, and the evolving metal/electrolyte interface also contribute to the observed growth behavior.

Here, “remote growth” refers to Li that becomes visible away from the initial W-probe contact. The operando sequences show how these features develop with time, but the visible position does not necessarily represent the original nucleation site. Depending on the local geometry, remote growth may involve continued deposition at an expanding Li/LLZTO interface, spreading or redistribution of existing Li, or Li emerging from a local opening. These processes are not mutually exclusive and may evolve together during continued polarization.

The operando measurements reveal two related aspects of spatially heterogeneous Li growth: the deposit can extend well beyond its initial contact, and the adjacent electrolyte can undergo measurable local displacement as growth proceeds. The newly recovered elemental comparison further identifies local Ta/Zr variation as a plausible material contributor. These observations connect the growth history, evolving contact geometry and local mechanical response, providing a basis for understanding localized degradation in garnet electrolytes.

## 4. Conclusions

Operando SEM revealed contact-confined and spatially extended Li growth on fractured and polished LLZTO surfaces. Time sequences on a polished pellet tracked the appearance, elongation, and branching of remote filamentary features. On a fractured surface, growth accompanied progressive local opening and material displacement. Another experiment followed the extension of an existing deposit as its shape and contact with the surface evolved. These observations describe distinct spatial growth histories under the individual test conditions.

Site-specific STEM-EDX revealed contrasting Ta and Zr signals near a region of extended growth, consistent with local compositional variation, while a separate contact-confined-growth region displayed a more uniform elemental pattern. Cryogenic 3D EDX provided a separate example of elemental nonuniformity in an uncycled region. These findings supply local chemical context for the operando observations and identify composition as a candidate factor to consider alongside contact geometry and surface condition.

Overall, this study links Li growth beyond a local electrode contact with evolving morphology and mechanical displacement in LLZTO. Combining time-resolved microscopy with local elemental characterization provides insight into how localized growth develops and interacts with the surrounding electrolyte. More broadly, the operando approach provides a useful way to identify localized degradation processes that are difficult to distinguish by post-mortem characterization alone.

## Data Availability

The original contributions presented in this study are included in the article/Supplementary Material. Further inquiries can be directed to the corresponding author.

## Supplementary Materials

The following supporting information can be downloaded at https://www.mdpi.com/article/10.3390/nano16211414/s1. Table S1: ICP-OES elemental concentrations of LLZTO powder; Table S2: ICP-OES-derived cation ratios and Ta substitution level; Figure S1: Low-magnification SEM images of polished and fractured LLZTO pellet surfaces; Figure S2: Grain-size analysis of the fractured LLZTO surface; Figure S3: XRD characterization of the LLZTO powder and comparison with the cubic garnet reference pattern; Figure S4: Image registration and quantitative analysis of local LLZTO displacement corresponding to Figure 2; Figure S5: Substrate-referenced analysis of remote Li filament growth corresponding to Figure 3; Figure S6: STEM-EDX characterization of an LLZTO region after contact-confined Li growth; Figure S7: Independent operando observation of extended Li growth on a polished LLZTO pellet; Video S1: Contact-confined Li growth on a fractured LLZTO surface; Video S2: Contact-confined Li growth on a polished LLZTO surface; Video S3: Li growth with local opening and displacement on a fractured LLZTO surface; Video S4: Remote filamentary Li growth on a polished LLZTO surface; Video S5: Extension of an existing Li deposit on a fractured LLZTO surface during a bias step; Video S6: Three-dimensional EDX reconstruction of La, Ta, and Zr distributions in uncycled LLZTO; Video S7: Independent observation of extended Li growth on a polished LLZTO surface.
