# Supplementary material for: Operando Visualization of Spatially Heterogeneous Lithium Growth on Ta-Doped LLZO
Source: Nanomaterials (Basel). 2026 Nov 3;16(21):1414. doi: 10.3390/nano16211414 (PMC13764881; doi:10.3390/nano16211414)
Supplement: Supplementary file 1 [file nanomaterials-16-01414-s001.zip › Supplementary Materials/Supplementary Materials.pdf]

Supplementary Materials

Operando Visualization of Spatially Heterogeneous Lithium Growth on Ta-Doped LLZO

Jinchun Guo and Xinwei Zhou*

School of Optoelectronic Engineering and Instrumentation Science, Dalian University of Technology, Dalian 116024, China

* Correspondence: zhouxinwei@dlut.edu.cn

The following information is included:

Table S1: ICP-OES elemental concentrations of LLZTO powder.

Table S2: ICP-OES-derived cation ratios and Ta substitution level.

Figure S1: Low-magnification SEM images of polished and fractured LLZTO pellet surfaces**.**

Figure S2: Grain-size analysis of the fractured LLZTO surface.

Figure S3: XRD characterization of the LLZTO powder and comparison with the cubic garnet reference pattern.

Figure S4: Image registration and quantitative analysis of local LLZTO displacement corresponding to Figure 2.

Figure S5: Substrate-referenced analysis of remote Li filament growth corresponding to Figure 3.

Figure S6: STEM-EDX characterization of an LLZTO region after contact-confined Li growth.

Figure S7: Independent operando observation of extended Li growth on a polished LLZTO pellet.

Video S1: Contact-confined Li growth on a fractured LLZTO surface. Corresponding to Figure 1b–d.

Video S2: Contact-confined Li growth on a polished LLZTO surface. Corresponding to Figure 1e–g.

Video S3: Li growth with local opening and displacement on a fractured LLZTO surface. Corresponding to Figure 2.

Video S4: Remote filamentary Li growth on a polished LLZTO surface. Corresponding to Figure 3.

Video S5: Extension of an existing Li deposit on a fractured LLZTO surface during a bias step. Corresponding to Figure 4.

Video S6: Three-dimensional EDX reconstruction of La, Ta, and Zr distributions in uncycled LLZTO.

Video S7: Independent observation of extended Li growth on a polished LLZTO surface. Corresponding to Figure S1.

Videos S1–S5, and S7 were compiled at 30 frames per second, with each frame corresponding to 1 s of experimental time; therefore, the videos are displayed at 30 times the real experimental rate. Video S6 is a three-dimensional EDX reconstruction and does not represent time-resolved operando data.

Two LLZTO powder aliquots (0.0376 and 0.0359 g) were independently weighed into cleaned polytetrafluoroethylene digestion vessels. Each aliquot was mixed with 3 mL HNO_3, 1 mL HCl and 0.5 mL HF and subjected to microwave-assisted digestion using an Anton Paar MW3000 system with a recorded temperature-control setting of 190 °C. After digestion, the solutions were diluted with ultrapure water and further diluted tenfold before analysis. Procedural blanks were prepared using the same protocol. Li, La, Zr and Ta were measured using a Thermo ICP-OES 6300 instrument in radial observation mode at an RF power of 1150 W. Concentrations are reported on a powder-mass basis (Table S1). Molar ratios and the Ta substitution level were calculated from these concentrations (Table S2).

Table S1. ICP-OES elemental concentrations of LLZTO powder.

Sample	Powder mass (g)	Li (mg kg^{-1})	La (mg kg^{-1})	Zr (mg kg^{-1})	Ta (mg kg^{-1})
Aliquot 1	0.0376	42,297.9	436,516.0	122,843.1	111,994.7
Aliquot 2	0.0359	44,206.1	431,894.2	131,554.3	120,111.4
**Mean**	**—**	**43,252.0**	**434,205.1**	**127,198.7**	**116,053.1**

Concentrations are expressed on a powder-mass basis. The two aliquots were digested independently (n = 2). Ta values incorporate the factor-of-ten conversion correction confirmed by the testing facility. Means are arithmetic averages of the individual aliquot results.

Table S2. ICP-OES-derived cation ratios and Ta substitution level.

Sample	Li	La	Zr	Ta	Zr + Ta	Ta/Zr	x
Aliquot 1	5.8183	3.0000	1.2855	0.5909	1.8764	0.4596	0.6298
Aliquot 2	6.1459	3.0000	1.3914	0.6405	2.0319	0.4603	0.6304
**Mean**	**5.9821**	**3.0000**	**1.3385**	**0.6157**	**1.9541**	**0.4600**	**0.6301**

Li, La, Zr and Ta ratios are normalized to La = 3. Molar amounts were calculated using atomic masses Li = 6.94, La = 138.90547, Zr = 91.224 and Ta = 180.94788 g mol^{-1}. The Ta substitution parameter is $x = 2n(Ta)/[n(Zr) + n(Ta)]$, assuming a total Zr/Ta occupancy of two sites. The La-normalized cation ratios are reported separately from this site-normalized x. Means are arithmetic averages of the individual aliquot values.

The two independent digestions give x = 0.6298 and 0.6304, corresponding to x ≈ 0.63.

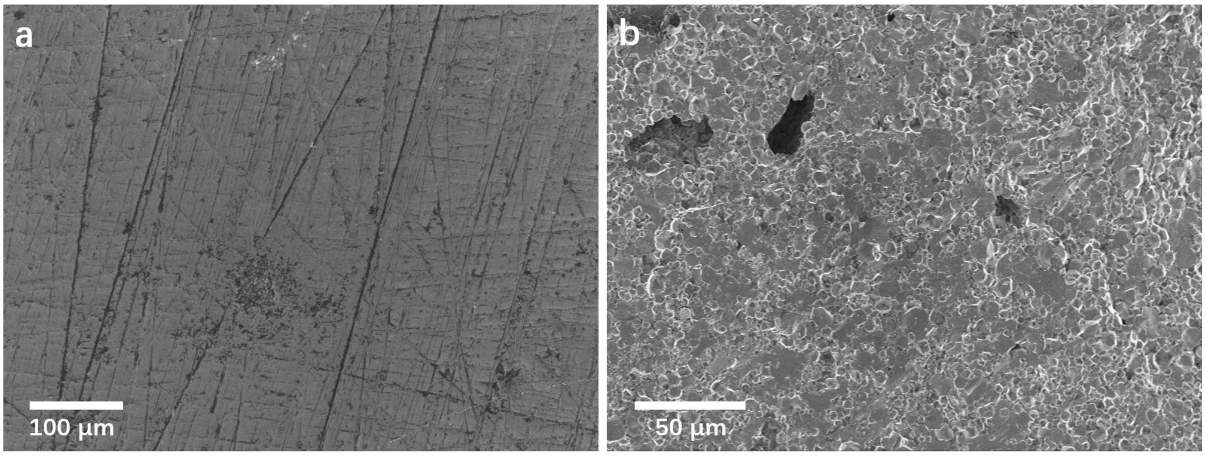

Figure S1. Low-magnification SEM characterization of LLZTO pellet surfaces.

(a) Polished LLZTO pellet surface showing the surface morphology after manual polishing. (b) Fractured LLZTO pellet surface showing the granular microstructure of the sintered ceramic.

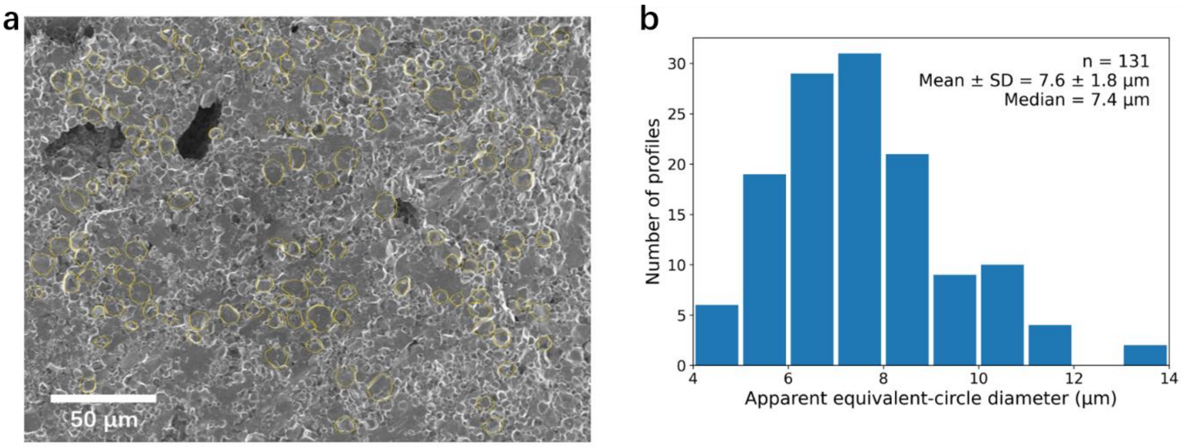

Figure S2. Grain-size analysis of the fractured LLZTO surface.

(a) Fractured-surface SEM image with selected grain profiles outlined for analysis. (b) Distribution of the apparent equivalent-circle diameters of the selected grains.

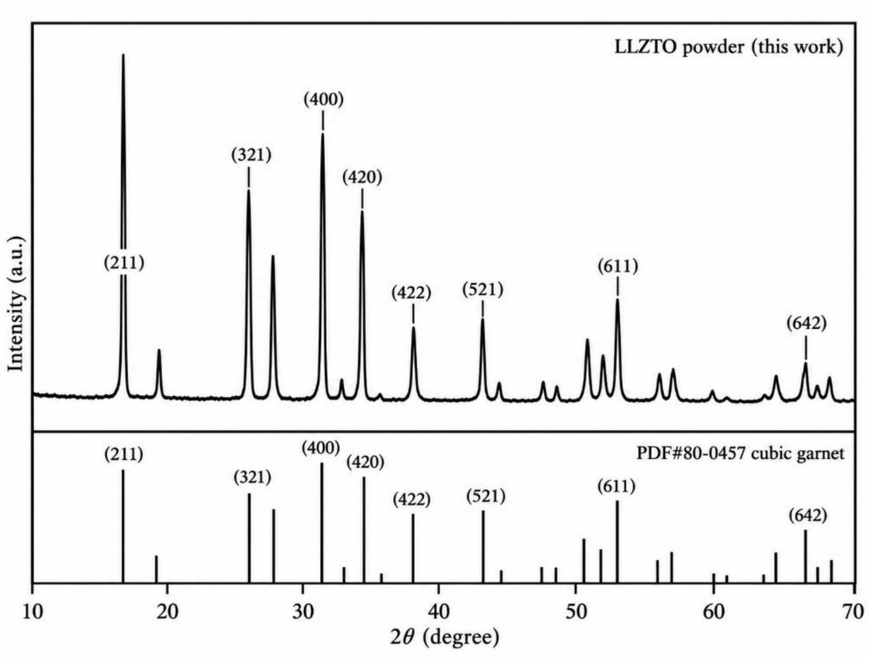

Figure S3. XRD characterization of the LLZTO powder.

Measured XRD pattern of the LLZTO powder compared with the reference pattern of cubic garnet $Li_5La_3Nb_2O_{12}$ (PDF#80-0457). The major diffraction peaks are consistent with the characteristic reflections of the cubic garnet structure.

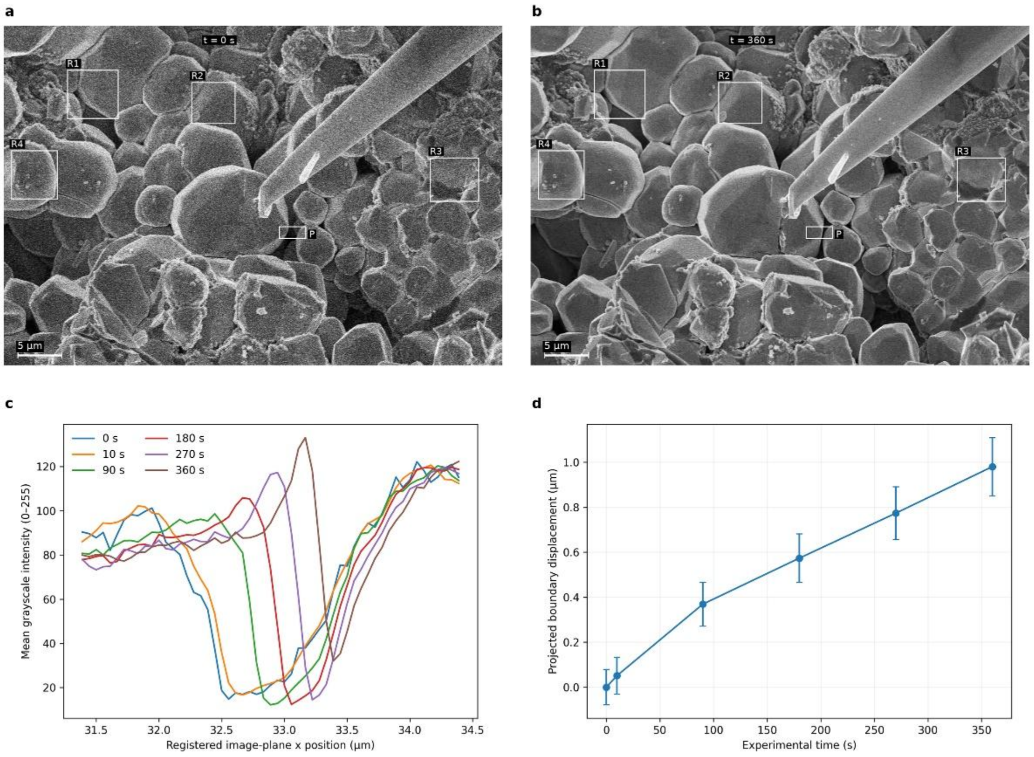

Figure S4. Image registration and quantitative analysis of local LLZTO displacement corresponding to Figure 2.

(a,b) Registered SEM images at 0 and 360 s showing the substrate reference regions and the

selected boundary region used for analysis. (c) Grayscale intensity profiles across the selected LLZTO boundary at different experimental times. (d) Projected displacement of the selected LLZTO boundary as a function of time. Error bars represent the estimated image-measurement uncertainty.

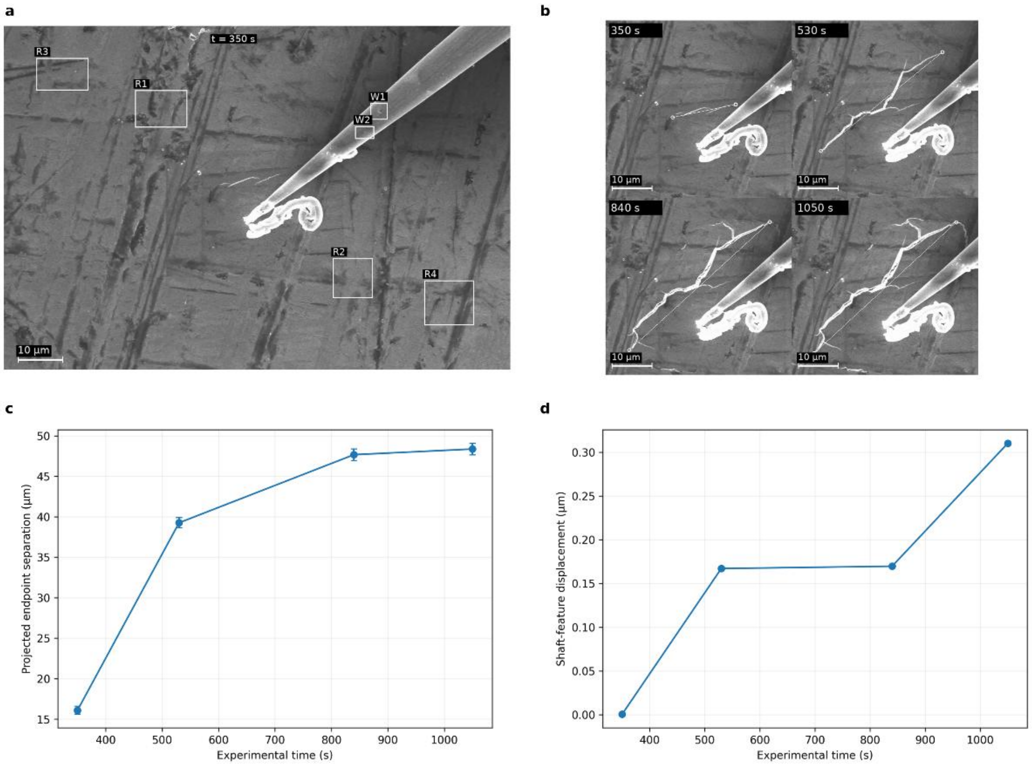

Figure S5. Substrate-referenced analysis of remote Li filament growth corresponding to Figure 3.

(a) SEM image at 350 s showing the substrate reference regions and the W-probe shaft features used for image registration and probe-motion analysis. (b) Selected endpoints of the principal remote Li filament at 350, 530, 840, and 1050 s. (c) Projected endpoint separation of the filament as a function of time. (d) Displacement of the tracked W-probe shaft features relative to the registered substrate.

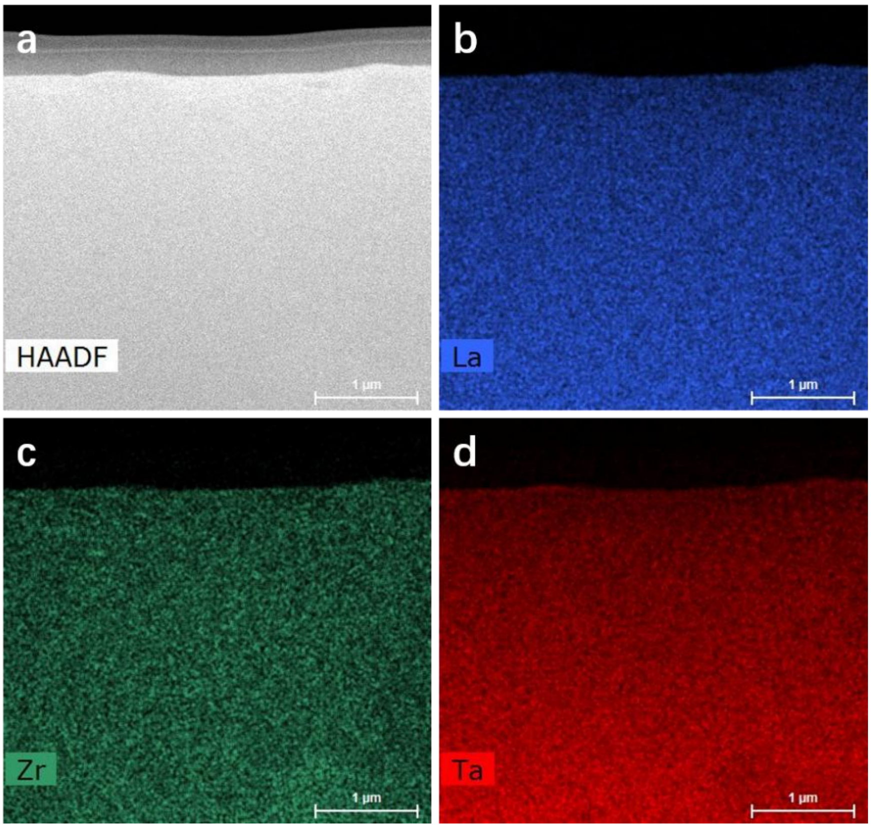

Figure S6. STEM-EDX characterization of an LLZTO region after contact-confined Li growth.
(a) HAADF-STEM image of the prepared LLZTO specimen. Corresponding STEM-EDX maps of (b) La, (c) Zr, and (d) Ta. Scale bars: 1 μm.

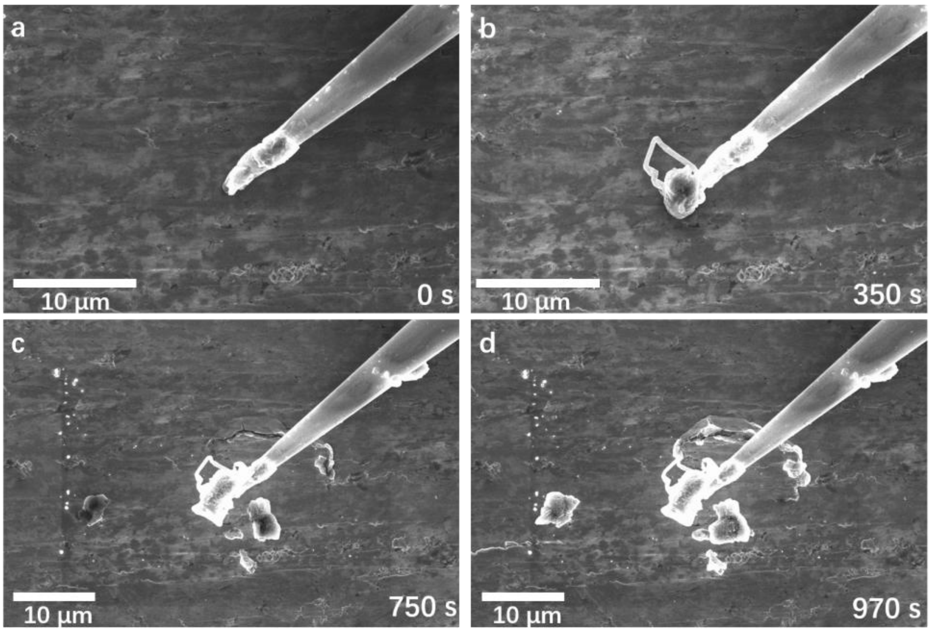

Figure S7. Independent operando observation of extended Li growth on a polished LLZTO pellet.

Operando SEM images recorded at (a) 0 s, (b) 350 s, (c) 750 s, and (d) 960 s under an applied bias of −0.5 V. The complete process is provided in Video S7. Scale bars: 10 μm.
